# Targeting myeloid-inflamed tumor with anti-CSF-1R antibody expands CD137+ effector T-cells in the murine model of pancreatic cancer

**DOI:** 10.1186/s40425-018-0435-6

**Published:** 2018-11-13

**Authors:** May Tun Saung, Stephen Muth, Ding Ding, Dwayne L. Thomas, Alex B. Blair, Takahiro Tsujikawa, Lisa Coussens, Elizabeth M. Jaffee, Lei Zheng

**Affiliations:** 1 Department of Oncology, Johns Hopkins University School of Medicine, Baltimore, MD USA; 2 The Sidney Kimmel Cancer Center, Johns Hopkins University School of Medicine, Baltimore, MD USA; 3 Department of Surgery, Johns Hopkins University School of Medicine, Baltimore, MD USA; 4 Department of Otolaryngology-Head and Neck Surgery, Kyoto Prefectural University of Medicine, Kyoto, Japan; 5 Department of Cell, Developmental & Cancer Biology, Oregon Health and Science University, Portland, OR USA; 6 Knight Cancer Institute, Oregon Health and Science University, Portland, OR USA; 7 The Skip Viragh Center for Pancreatic Cancer Research and Clinical Care, Johns Hopkins University School of Medicine, Baltimore, MD USA; 8 Pancreatic Cancer Precision Medicine Program, Johns Hopkins University School of Medicine, Baltimore, MD USA; 9 The Bloomberg-Kimmel Institute for Cancer Immunotherapy, Johns Hopkins University School of Medicine, Baltimore, MD USA

**Keywords:** Pancreatic ductal adenocarcinoma, Lymphoid aggregates, Cytotoxic T-cells, Tumor associated macrophages, Dendritic cells, PD-1, CSF-1R, CD137, GVAX, Interferon-γ

## Abstract

**Background:**

The pancreatic cancer vaccine, GVAX, induces novel lymphoid aggregates in the otherwise immune quiescent pancreatic ductal adenocarcinoma (PDAC). GVAX also upregulates the PD-1/PD-L1 pathway, and a pre-clinical model demonstrated the anti-tumor effects of combination GVAX and anti-PD-1 antibody therapy (GVAX/αPD-1). Resistance to GVAX was associated with an immune-suppressive myeloid cell infiltration, which may limit further therapeutic gains of GVAX/αPD-1 therapy. The expression of CSF-1R, a receptor important for myeloid cell migration, differentiation and survival, and the effect of its therapeutic blockade in the context of GVAX in PDAC has not been investigated.

**Methods:**

Lymphoid aggregates appreciated in 24 surgically resected PDAC from patients who received one dose of neoadjuvant GVAX were analyzed with multiplex immunohistochemistry. Flow cytometry analysis of tumor infiltrating T-cells in a murine model of PDAC was performed to investigate the therapeutic effects and mechanism of anti-CSF-1R/anti-PD-1/GVAX combination immunotherapy.

**Results:**

High CSF-1R expression in resected PDAC from patients who received neoadjuvant GVAX was associated with a higher myeloid to lymphoid cell ratio (*p* < 0.05), which has been associated with poorer survival. This higher CSF-1R expression was associated with a higher intra-tumoral infiltration of immature dendritic cells (*p* < 0.05), but not mature dendritic cells (*p* = 0.132). In the pre-clinical murine model, administering anti-CSF-1R antibody prior to and after GVAX/αPD-1 (“pre/post-αCSF-1R + αPD-1 + GVAX”) enhanced the survival rate compared to GVAX/αPD-1 dual therapy (*p* = 0.005), but administering anti-CSF-1R only before GVAX/αPD-1 did not (*p* = 0.41). The “pre/post-αCSF-1R + αPD-1 + GVAX” group also had higher intra-tumoral infiltration of PD-1 + CD8+ and PD-1 + CD4+ T-cells compared to αPD-1/GVAX (*p* < 0.001). Furthermore, this regimen increased the intra-tumoral infiltration of PD-1 + CD137 + CD8+, PD-1 + CD137 + CD4+ and PD-1 + OX40 + CD4+ T-cells (*p* < 0.001). These PD-1 + CD137 + CD8+ T-cells expressed high levels of interferon-γ (median 80–90%) in response to stimulation with CD3/CD28 activation beads, and this expression was higher than that of PD-1 + CD137-CD8+ T-cells (*p* < 0.001).

**Conclusions:**

The conversion of exhausted PD-1+ T-cells to CD137+ activated effector T-cells may contribute to the anti-tumor effects of the anti-CSF-1R/anti-PD-1/GVAX combination therapy. Anti-CSF-1R antibody with anti-PD-1 antibody and GVAX have the potential be an effective therapeutic strategy for treatment of PDAC.

**Electronic supplementary material:**

The online version of this article (10.1186/s40425-018-0435-6) contains supplementary material, which is available to authorized users.

## Background

Pancreatic ductal adenocarcinoma (PDAC) is a devastating disease with a 5-year survival rate of 8% for all stages despite the availability of treatment with chemotherapy, radiation and/or surgery [1]. The survival decreases to 3% for patients with late stage disease [1]. Immunotherapy has shown few clinical responses in PDAC despite clinical success in other cancers [2–5]. Resistance to immunotherapy has in part been attributed to an immune quiescent tumor microenvironment (TME). The presence of increased anti-tumor effector T-cells may improve prognosis, but these effectors cells are rarely appreciated in PDAC [6, 7]. Additionally, when infiltrating immune cells are present, they tend to be immunosuppressive, such as regulatory T-cells, immature dendritic cells, myeloid-derived suppressor cells (MDSCs) and tumor-associated macrophages (TAMs) [8].

To induce infiltration of immune cells into the PDAC, a GM-CSF (granulocyte-macrophage colony-stimulating factor) secreting pancreatic cancer vaccine, GVAX, has been employed [3, 4, 9–11]. A single dose of neoadjuvant GVAX with or without immunomodulatory doses of cyclophosphamide induced the formation of tertiary lymphoid structures within two weeks of administration in 85% of vaccinated patients, whereas organized lymphoid structures were not present in unvaccinated patients (ClinicalTrials.gov identifier: NCT007272441) [3]. These tertiary lymphoid structures had organized T-cell and B-cell zones, germinal centers, lymphatic vessels and the presence of cytokines involved in lymphoid neogenesis [3]. The presence of similar lymphoid structures in immunotherapy-naïve patients has been associated with improved survival, and indeed patients with an overall survival of over 3 years were more likely to have developed lymphoid aggregates after GVAX [3, 12]. However, there were still patients who survived less than 1.5 years despite having developed organized lymphoid structures after GVAX treatment [3]. Further analysis of these lymphoid structures with multiplex immunohistochemistry (IHC) demonstrated that patients with high myeloid cell infiltration had lower survival compared to those with low myeloid cell infiltration, despite high lymphoid cell densities in both groups [13]. High myeloid and CD68+ infiltration into tumors has been associated with poor survival in many studies [14–17]. Thus, targeting myeloid cells may further enhance anti-tumor activity of vaccine therapy. Of particular interest would be to target the colony-stimulating factor 1-receptor (CSF-1R) within this patient cohort. CSF-1 and its receptor, CSF-1R, regulate the migration, differentiation and survival of macrophages and other myeloid cells [18, 19]. PDAC tumors often express high levels of CSF-1 compared to normal tissue, and CSF-1R can be detected in the tumor stroma [20, 21]. High CSF-1R expression in PDAC has also been associated with worse prognosis compared to low CSF-1R expression [20, 21]. Inhibition of CSF-1R can lead to preferential TAM depletion and modulate the remaining myeloid cells toward a more anti-tumor phenotype and away from pro-tumor properties [19–21]. The combination of anti-CSF-1R and anti-PD-1 antibodies promotes anti-tumor activity in pre-clinical models, and this combination is actively investigated in several clinical trials for many types of tumors, including pancreatic cancer [20, 22–24]. It is encouraging that one clinical trial has already reported that dual PD-1 and CSF-1R blockade may provide durable clinical benefit in patients with chemotherapy-treated and immunotherapy-naïve advanced pancreatic cancer (ClinicalTrials.gov identifier: NCT02526017) [25].

In our prior study, GVAX induced both markers of early T-cell activation and PD-L1 and PD-1 expression within the lymphoid aggregates present in the post-vaccination patients, suggesting that vaccine therapy can prime PDAC for PD-1/PD-L1 inhibition therapies [3]. The survival benefit of adding GVAX to PD-1 blockade was demonstrated in a murine model of liver metastatic pancreatic cancer [26]. We propose that the addition of anti-CSF-1R antibody to the combination of GVAX therapy and anti-PD-1 antibody can improve the survival outcome of PDAC by further altering the myeloid population and creating a more anti-tumor immune environment.

## Study methods

### Human pancreatic ductal adenocarcinoma multiplex IHC

The effect of CSF-1R expression level on the tumor-infiltrating immune cells in human PDAC tumor specimens was investigated based on the data generated from our previous multiplex immunohistochemistry analysis [13]. We analyzed 24 formalin-fixed paraffin-embedded specimens obtained after surgical resection of the tumor two weeks after one neoadjuvant intradermal administration of GVAX alone or in combination with immune modulatory doses of cyclophosphamide in patients with resectable PDAC (ClinicalTrials.gov identifier: NCT00727441) [3]. These 24 specimens had novel intratumoral lymphoid aggregates that were not present prior to GVAX administration [13]. The median cell density of CSF-1R expression was used as the cut off between high CSF-1R expression (higher than 14.5 cells/mm^2^) and low CSF-1R expression (lower than 14.5 cell/mm^2^), and the effect the level of CSF-1R expression had on the infiltration of immune cells into the PDAC was analyzed. Lymphoid cells were CD45+ and CD3+ or CD20+ or CD56+, and myeloid markers were CD45+ and CD3-CD20-CD56- [13]. Within the myeloid cells, myelomonocytic cells were identified as CD66b-Tryptase-CD68 + CSF1R-, immature dendritic cells were identified as CD66b-Tryptase-MHC class II + DC-SIGN+CD83- and mature dendritic cells were identified as CD66b-Tryptase-MHC class II + CD83+ [13]. All cell densities were measured as cells positive for marker per mm^2^. Cell densities (cells/mm^2^) were used to calculated ratios between cell types.

### Cell lines and medium

KPC is a previously established PDAC tumor cell line that was derived from transgenic mice in a C57Bl6 background with tissue-specific Kras and p53 knock-in mutations [27]. KPC cells were maintained at 37 °C in 5% CO_2_ with RMPI 1640 media (Life Technologies) supplemented with 10% heat-inactivated fetal bovine serum (HI-FBS, Benchmark), 1% penicillin/streptomycin (pen/strep, Life Technologies), 1% MEM-NEAA, −(Minimum Essential Medium-Non-Essential Amino Acids, Life Technologies), 1% sodium pyruvate (Sigma) and 1% L-glutamine (Life Technologies). B78H1 is an MHC (major histocompatibility complex) class I negative murine fibroblast cell line engineered to secrete GM-CSF. B78H1 cells were maintained at 37 °C in 5% CO_2_ with RMPI 1640 media supplemented with 10% HI-FBS, 1% pen/strep and 0.5% % L-glutamine. Harvested tumor-infiltrating immune cells were processed in T-cell media, which consisted of RPMI 1640 media supplemented with 10% HI-FBS, 1% pen/strep, 1% HEPES (Life Technologies), 1% MEM-NEAA, 0.5% L-glutamine and 0.05% 2-mercaptoethanol (Sigma).

### Mice tumor inoculation and treatment regimens

Seven- to eight-week old C57Bl6 mice were purchased from Harlan Laboratories or Jackson Laboratories, and maintained in accordance with the Institutional Animal Care and Use Committee (IACUC) guidelines. When the mice were eight- to ten-weeks old, KPC tumor cell inoculation was performed via the hemispleen technique on day 0 as previously described [28]. Briefly, after anesthetizing the mice, the spleen was eviscerated, clipped and divided in half. 2 × 10^5^ KPC tumor cells in 100 μL of PBS (Gibco) were carefully injected into half the spleen, and flushed with 150 μL of PBS. The half of the spleen through which the tumor cells were injected was then removed to avoid leaving residual tumor cells external to the liver. The peritoneum and the skin were then sutured.

Anti-mouse PD-1 G1-D265A antibodies (clone 9H2, Bristol-Myers-Squibb) were administered intraperitoneally (IP) at 100 μg (5 mg/kg) per dose on days 7 and 11, and murine GVAX vaccine was administered subcutaneously (SC) on day 7. GVAX vaccine was prepared by combining 1 × 10^6^ KPC cells and 1 × 10^6^ B78H1 cells in PBS to make a total combined cell concentration of 20 × 10^6^ cells/mL. The cell suspension was then irradiated at 50 Gy and administered SC in 3 locations (bilateral flanks and one of the upper limbs) at 100 μL per injection (6 × 10^6^ cells injected in total per mouse). For “Pre/Post-αCSF-1R” dosing, rat anti-mouse CSF-1R G2a antibodies (clone AFS98, BioXcell) were administered IP at 1.5 mg (75 mg/kg) per dose on days 1, 4, 8 and 11. For “Pre-αCSF-1R” dosing, rat anti-mouse CSF-1R G2a antibodies were administered IP at 1.5 mg per dose on days 1 and 4. For “Post-αCSF-1R” dosing, rat anti-mouse CSF-1R G2a antibodies were administered IP at 1.5 mg per dose on days 8 and 11.

### Survival studies

For survival studies, the mice were monitored at least once a day. Mice with signs of distress, including hunched posture and lethargy, were euthanized and considered to have reached the “survival” endpoint in accordance to IACUC guidelines.

### Analysis of liver metastasis infiltrating immune cells

Mice were sacrificed and liver metastasis infiltrating immune cells were harvested on day 14, which was one week after the dose of GVAX. Each liver was mechanically processed and suspended sequentially first through a 100-μm nylon filter and then through a 40-μm nylon filter, and brought to a volume of 25 mL in T-cell media. The cell suspensions were centrifuged at 1500 rpm for 5 min. The supernatant was aspirated, and the cell pellets were resuspended in 4 mL of ACK lysing buffer (Quality Biological). After 2 min in ACK buffer, the lysing was quenched by adding T-cell media up to a volume of 50 mL. The samples were then centrifuged at 1500 rpm for 5 min, and the supernatant was aspirated. The cell pellet was resuspended in 6 mL of 80% Percoll (GE Healthcare), then overlaid with 6 mL of 40% Percoll, and centrifuged at 3200 rpm for 25 min without braking. The leukocyte layer was removed and resuspended in 50 mL of T-cell media, washed twice and re-suspended in PBS.

### Cell surface staining and flow cytometry

Following the isolation of liver metastasis pancreatic tumor infiltrating immune cells, the cells from mice from the same treatment group were pooled and resuspended in PBS at a concentration of 2 × 10^7^ cells/mL. For each treatment group, triplicates of this pooled cell suspension were placed into wells of a 96-well plate at a volume of 100 μL per well. The cells in each well were stained with Live-Dead Aqua (Invitrogen) for 30 min on ice, washed twice with PBS and then blocked with rat anti-mouse Fc antibody (CD16/CD32, clone 2.4G2, BD Biosciences) in FACs buffer for 10 min on ice. The FACs buffer consisted of HBSS (Sigma) with 2% bovine calf serum (Sigma), 0.1% sodium azide (Sigma) and 0.1% HEPES. After Fc blocking, the cells were stained for the following anti-mouse fluorophores for 1 h on ice: CD45 PerCP-Cy5.5 (clone 30-F11, BD Biosciences), CD4 APC-H7 (clone GK1.5, BD Biosciences), CD8a PE/Cy7 (clone 53–6.7, Biolegend), PD-1 FITC (clone RMP1–30, eBioscience), and CD137 APC (clone 17B5, eBioscience) or OX40 APC (clone OX-86, Biolegend).

The cells were then washed twice and resuspended in FACs buffer, and flow cytometry was performed using the Gallios flow cytometer (Beckman Coulter).

### Intracellular staining for interferon-γ and flow cytometry

The isolated liver metastasis pancreatic tumor infiltrating immune cells of each mouse were enriched for CD8+ T-cells by negative isolation as per the manufacturer’s protocol (Dynabeads Untouched Mouse CD8 cells, Life Technologies). The isolated CD8+ T-cells were incubated with CD3/CD28 stimulation beads (Dynabeads Mouse T-activator CD3/CD28, Life Technologies) according to manufacturer’s protocol at 1:1 cell to bead ratio in T-cell media for 7 h at 37 °C in 5% CO_2_ before GolgiPlug (BD Biosciences) was added at 1:1000 volume ratio, after which the cells were incubated for 5 more hours at 37 °C in 5% CO_2._ After a total incubation time of 12 h, the beads were removed as per manufacturer’s protocol and washed 2 times with PBS. The cells from each mouse were kept separate, and were not pooled. The cells were then stained with Live-Dead Aqua (Invitrogen), CD8a PE/Cy7 (clone 53–6.7, Biolegend), PD-1 FITC (clone RMP1–30, eBioscience) and CD137 eFluor450 (clone 17B5, eBioscience) as described above. The cells were washed twice with PBS, resuspended and incubated in Fixation/Permeabilization buffer (eBioscience) for 30 min at 4 °C. The cells were washed twice with Permeabilization buffer (eBioscience), and then incubated with interferon-γ APC (clone XMG1.2, eBioscience) for 30 min on ice. The cells were washed in twice with Permeabilization buffer and resuspended in FACs buffer. IgGκ1 APC isotype control (clone eBRG1, eBioscience) was used for interferon-γ (IFN-γ).

### Statistical analysis

For comparisons of cell density and cell density ratios between high and low CSF-1R expression in the human PDAC samples, the mean values were analyzed using the unpaired t-test. Survival analysis in murine experiments were performed using the univariate Cox model. For comparison of cell number, percentage and cytokine expression in murine experiments between groups, the mean values were analyzed using the unpaired one-way ANOVA with Bonferroni *p*-value adjustment for multiple comparisons. All tests were two-tailed, and *P* < 0.05 was considered statistically significant.

## Results

### High CSF-1R expression after GVAX administration is associated with an immunosuppressive TME in human PDAC

**Fig. 1:**
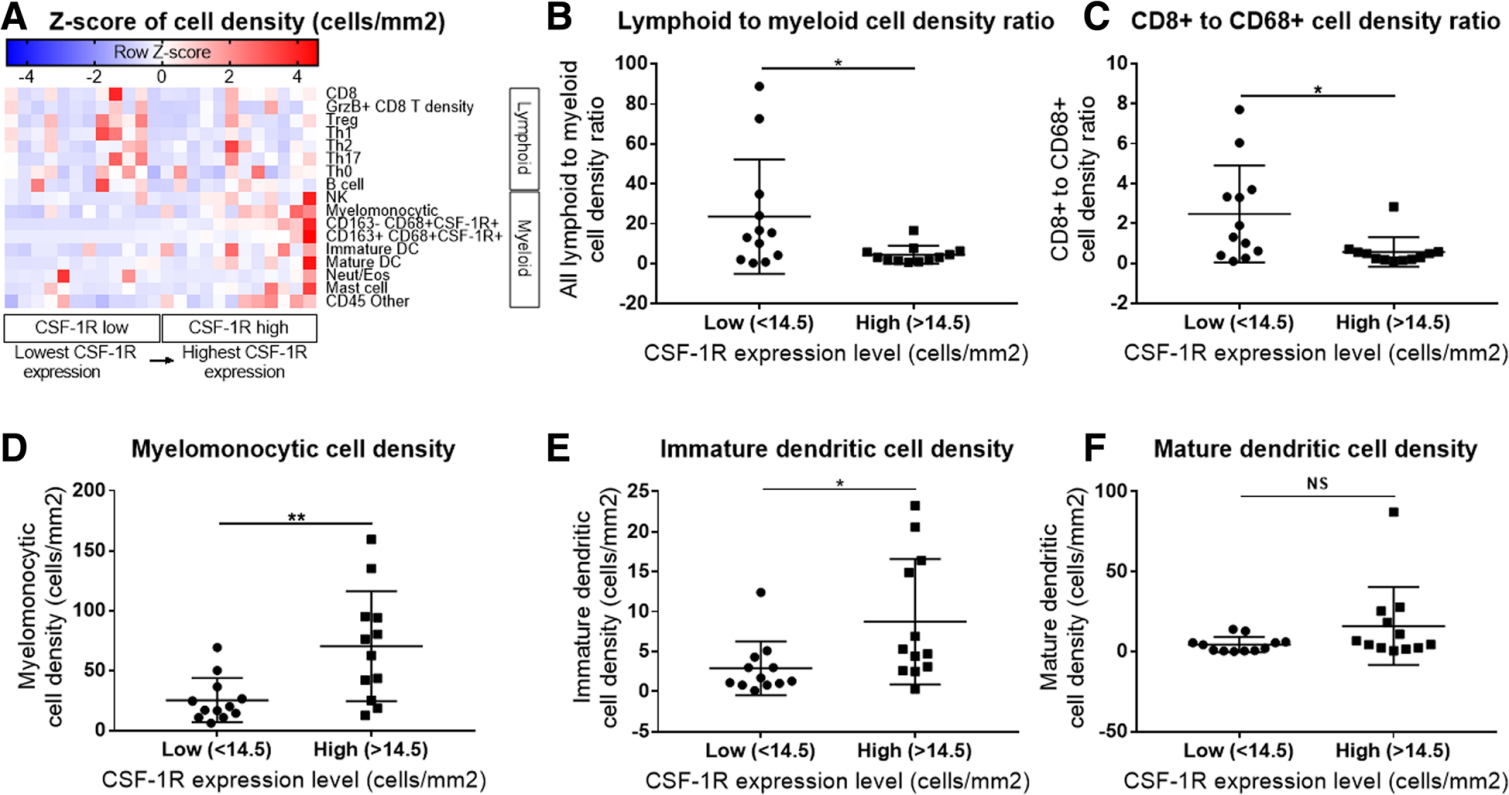
High CSF-1R expression is associated with an immunosuppressive TME. 24 formalin-fixed paraffin-embedded human PDAC specimens obtained after surgical resection of the tumor two weeks after one neoadjuvant intradermal administration of GVAX alone or in combination with immune modulatory doses of cyclophosphamide were analyzed using multiplex immunohistochemistry. The median cell density of CSF-1R expression was used as the cut off between high CSF-1R expression (higher than 14.5 cells/mm2) and low CSF-1R expression (lower than 14.5 cell/mm2), and the effect the level of CSF-1R expression had on the PDAC-infiltrating immune cells was analyzed. **a** Heat map of the z-score of CSF-1R positive cell density (x-axis, cells/mm2) of various tumor infiltrating immune cells (rows, with lymphoid cells in the upper rows and myeloid cells in the lower rows). **b** Lymphoid to myeloid cell and (**c**) CD8+ to CD68+ cell density ratio (cells/mm2 to cells/mm2) within lymphoid aggregates with low versus high CSF-1R expression. **d** Myelomonocytic cell, (**e**) immature dendritic cell and (**f**) mature dendritic cell density within lymphoid aggregates with low versus high CSF-1R expression. Lymphoid cells were CD45+ and CD3+ or CD20+ or CD56+, and myeloid markers were CD45+ and CD3-CD20-CD56-. Myelomonocytic cells were defined as CD45 + CD3-CD20-CD56-CD66b-Tryptase-CD68 + CSF1R-. Immature DCs were defined as CD45 + CD3-CD20-CD56-CD66b-Tryptase-MHC class II + DC-SIGN+CD83-, and mature DCs were defined as CD45 + CD3-CD20-CD56-CD66b-Tryptase-MHC class II + CD83+. The error bars represent mean with standard deviation. * *p* < 0.05; ** *p* < 0.01; NS, non-significant

We first investigated the effect of high versus low CSF-1R expression within the lymphoid aggregates that formed in the human PDAC tumors after a single neoadjuvant dose of GVAX. As anticipated, low CSF-1R expression was associated with high lymphoid to myeloid cell ratio (Fig. 1a and b) and high CD8+ T-cell to CD68+ cell ratio (Fig. 1c). High CSF-1R expression was associated with a higher infiltration of myelomonocytic cells and immature dendritic cells (DCs) into the lymphoid aggregates (Fig. 1d and e), both of which are associated with immune-suppressive environments. [29–31] However, the level of CSF-1R expression did not have an effect on the infiltration of mature DCs (Fig. 1f). These findings suggest that CSF-1R expression is preferentially associated with immunosuppressive myeloid cells in vaccine-primed PDAC and thus a potential target of immunotherapy for PDAC.

### Anti-CSF-1R antibody in combination with GVAX and anti-PD-1 antibody improves survival in a metastatic PDAC murine tumor model

**Fig. 2:**
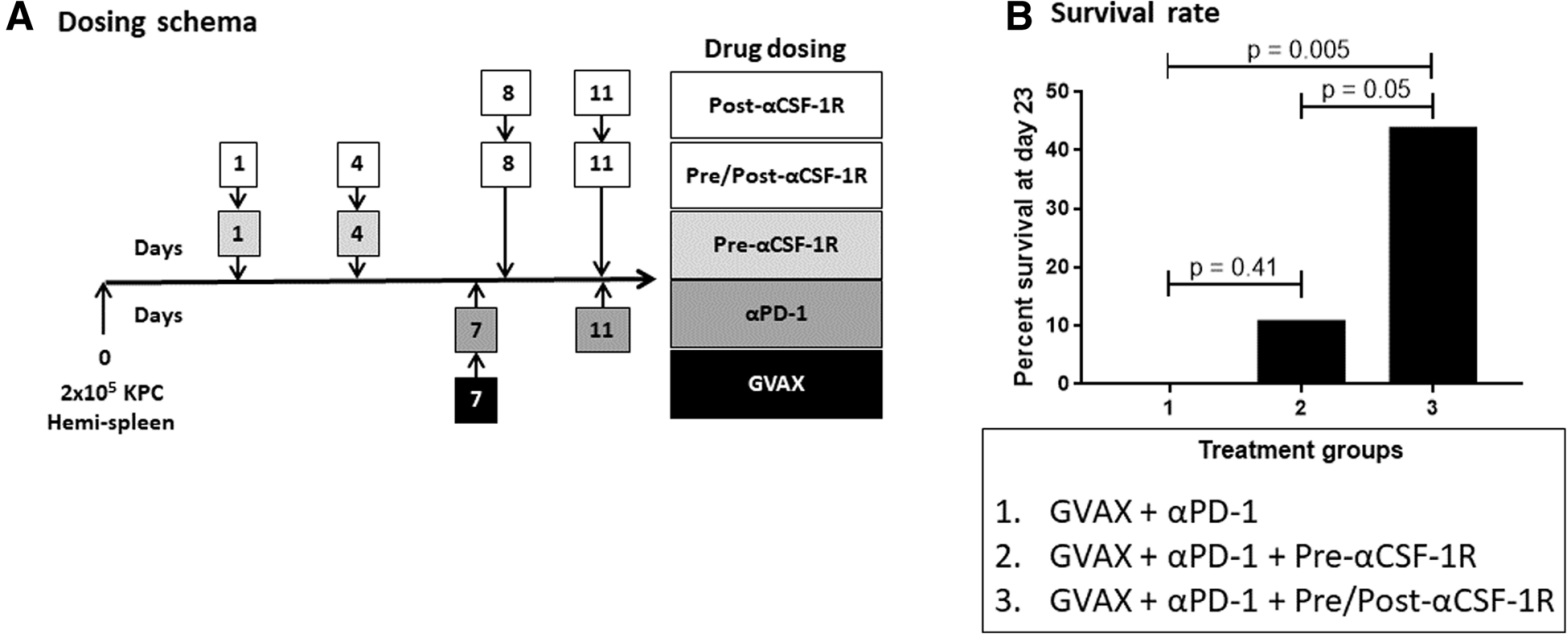
The addition of αCSF-1R to GVAX therapy and αPD-1 improves survival rate in a liver metastatic pancreatic cancer murine model. **a** 2 × 10^5^ KPC tumor cells were inoculated via a hemispleen surgery on day 0, and mice were treated with GVAX and αPD-1 as indicated, and αCSF-1R was dosed in three different ways as shown. **b** Treated mice were followed for survival, and the percent survival at day 23 was calculated. No mice survived beyond day 25. GVAX + αPD-1 (*n* = 10), GVAX + αPD-1 + Pre-αCSF-1R (*n* = 9), GVAX + αPD-1 + Pre/Post-αCSF-1R (*n* = 9)

Given the effects of CSF-1R expression on the human PDAC tumor immune microenvironment after receiving GVAX, we hypothesized that the addition of anti-CSF-1R antibody (αCSF-1R) to GVAX and anti-PD-1 antibody (αPD-1) could further improve survival outcomes. We used the previously reported murine model of liver metastatic pancreatic cancer, where the KPC tumor cells were injected into half the spleen on day 0 to generate liver metastases, and this half of the spleen was removed to avoid residual tumor cells external to the liver [26, 28]. The mice were treated with one dose of GVAX on day 7, and αPD-1 was given on days 7 and 11 (Fig. 2a). We found that beginning αCSF-1R treatment after GVAX administration did not enhance the anti-tumor effect of GVAX and αPD-1 combination therapy in this mouse model (data not shown). Therefore, αCSF-1R was subsequently dosed in two different schedules to investigate if giving it before GVAX vaccination affected the survival outcome. One group of mice received αCSF-1R on days 1 and 4 prior to GVAX vaccination (regimen called “Pre-αCSF-1R”, Fig. 2a); and another group of mice received αCSF-1R on days 1, 4, 8 and 11, which was prior to and after GVAX vaccination (regimen called “Pre/Post-αCSF-1R”, Fig. 2a). The combination treatment of GVAX + αPD-1 + Pre/Post-αCSF-1R improved the day 23% survival compared to GVAX + αPD-1 only (44% vs. 0%, HR 0.14, 95% CI, 0.04–0.56, Fig. 2b). There was a trend towards a survival advantage with the GVAX + αPD-1 + Pre/Post-αCSF-1R treatment regimen over the GVAX + αPD-1 + Pre-αCSF-1R regimen, but this was not statistically significant (44% vs. 11%, HR 0.31, 95% CI, 0.10–0.98, Fig. 2b). There was no statistical difference in survival between the GVAX + αPD-1 + Pre-αCSF-1R and GVAX + αPD-1 regimens (11% vs. 0%, HR 0.67, 95% CI, 0.26–1.73, Fig. 2b). Because only one cycle of GVAX and αPD-1 treatment was given, none of the mice in any of these treatment groups survived beyond day 25.

### Anti-CSF-1R antibody in combination with GVAX and anti-PD-1 antibody increases PD-1 + CD137+ T-cells in the TME

**Fig. 3:**
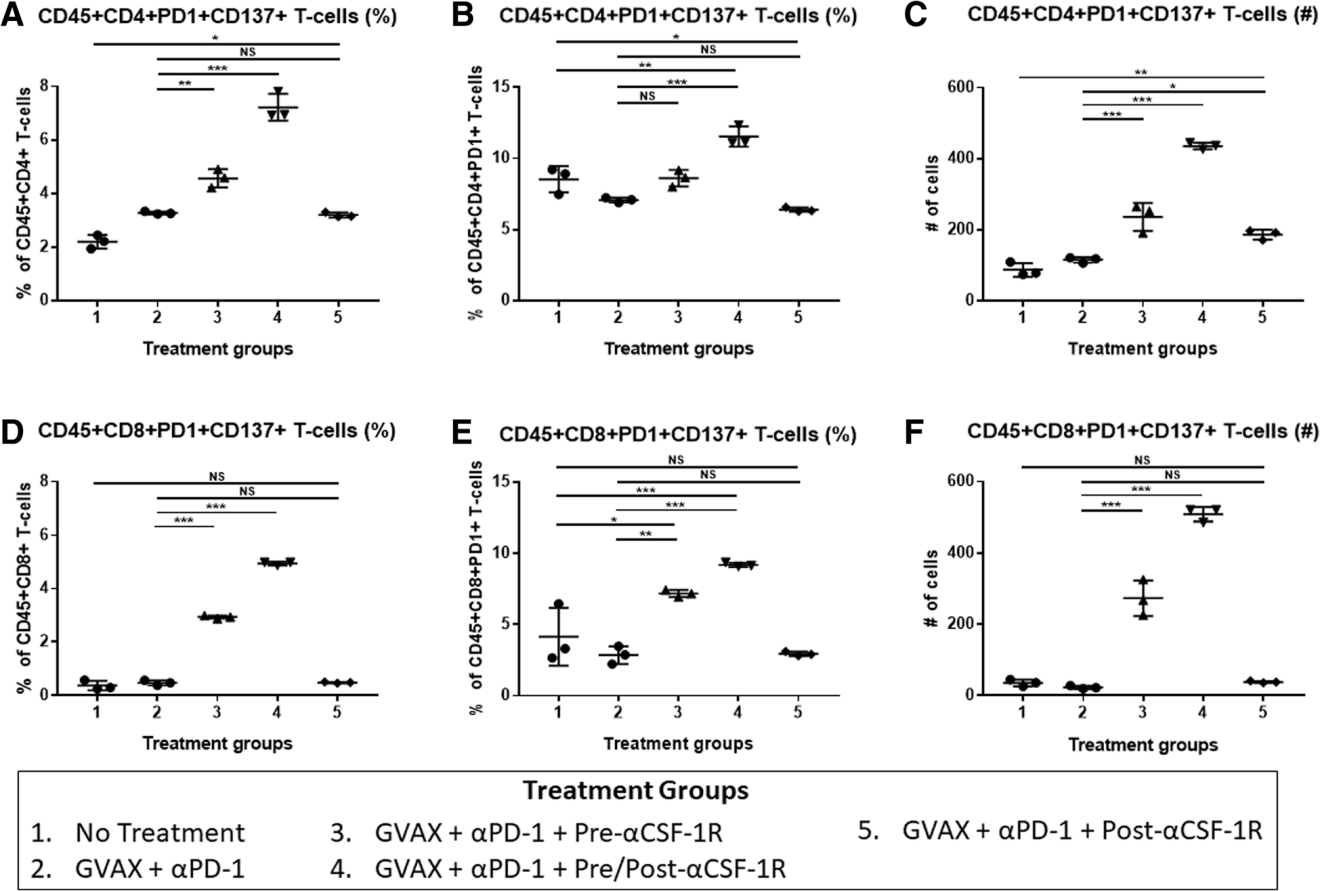
The PD-1 + CD137+ CD8+ T-cells increase with the addition of αCSF-1R to GVAX therapy and αPD-1. Liver metastatic KPC tumor-bearing mice were sacrificed on day 14 after receiving treatment, and flow cytometry analysis was performed on the isolated tumor infiltrating immune cells. The percentage of PD-1 + CD137+ cells within the (**a**) CD45 + CD4+ and (**d**) CD45 + CD8+ T-cell populations. The proportion of cells expressing CD137 amongst (**b**) CD45 + CD4 + PD-1+ and (**e**) CD45 + CD8 + PD-1+ T-cells. The number of (**c**) CD45 + CD4 + PD-1 + CD137+ and (**f**) CD45 + CD8+ PD-1 + CD137+ T-cells. *N* = 3 for each treatment group, and the isolated immune cells from mice from the same treatment group were pooled and measured in triplicates. *p < 0.05; **p < 0.01; ***p < 0.001; NS, non-significant

To understand the mechanistic basis of the enhanced anti-tumor activity of αCSF-1R (Fig. 2b), we performed flow cytometry analysis of the liver metastasis infiltrating T-cells one week after GVAX administration in the same liver metastatic pancreatic cancer murine model, with day 0 as the day of KPC tumor cell inoculation. All of the treated mice received αPD-1 on days 7 and 11, and GVAX on day 7 (Fig. 2a). αCSF-1R was administered in 3 different schedules to investigate its effect on the T-cell population (Fig. 2a), and the mice were sacrificed and the livers harvested on day 14. This analysis demonstrated that the addition of αCSF-1R both before and after the GVAX vaccination not only induced greater numbers of PD-1+ CD4+ or CD8+ tumor infiltrating T-cells in the “GVAX + αPD-1 + Pre/Post-αCSF-1R” group (Additional file 1: Figure S1A-D), but also increased the number and percentage of PD-1 + CD137+ T-cells (Fig. 3a-f, Additional file 2: Figure S2D). Up to 10% of the PD-1+ CD8+ T-cells expressed CD137 in the “GVAX + αPD-1 + Pre/Post-αCSF-1R” group, which was more than 2 or 3 times the average level of expression in the non-treatment and GVAX + αPD-1 groups, respectively (Fig. 3e). The number of infiltrating PD-1 + CD137+ CD4+ T-cells increased by about a factor of 4 (Fig. 3c) and increased by a factor of more than 20 for PD-1 + CD137+ CD8+ T-cells (Fig. 3f) in the “GVAX + αPD-1 + Pre/Post-αCSF-1R” group compared to the GVAX + αPD-1 group. Administering αCSF-1R only before the GVAX vaccination (GVAX + αPD-1 + Pre-αCSF-1R group) had a modest and less robust effect on increasing PD-1 + CD137+ T-cells. In contrast, the “GVAX + αPD-1 + Post-αCSF-1R” group did not significantly increase PD-1 + CD137+ T cells compared to the GVAX + αPD-1 group, except for slightly increasing the number of PD-1 + CD137+ CD4+ T-cells (Fig. 3c). These results suggest that αCSF-1R can enhance the expression of the CD137 activation marker in PD-1+ T-cells; however, the administration sequence of αCSF-1R, GVAX and αPD-1 is important for this activity of αCSF-1R.

### Anti-CSF-1R antibody in combination with GVAX and anti-PD-1 antibody increases PD-1 + OX40+ CD4+ T-cells in the TME

**Fig. 4:**
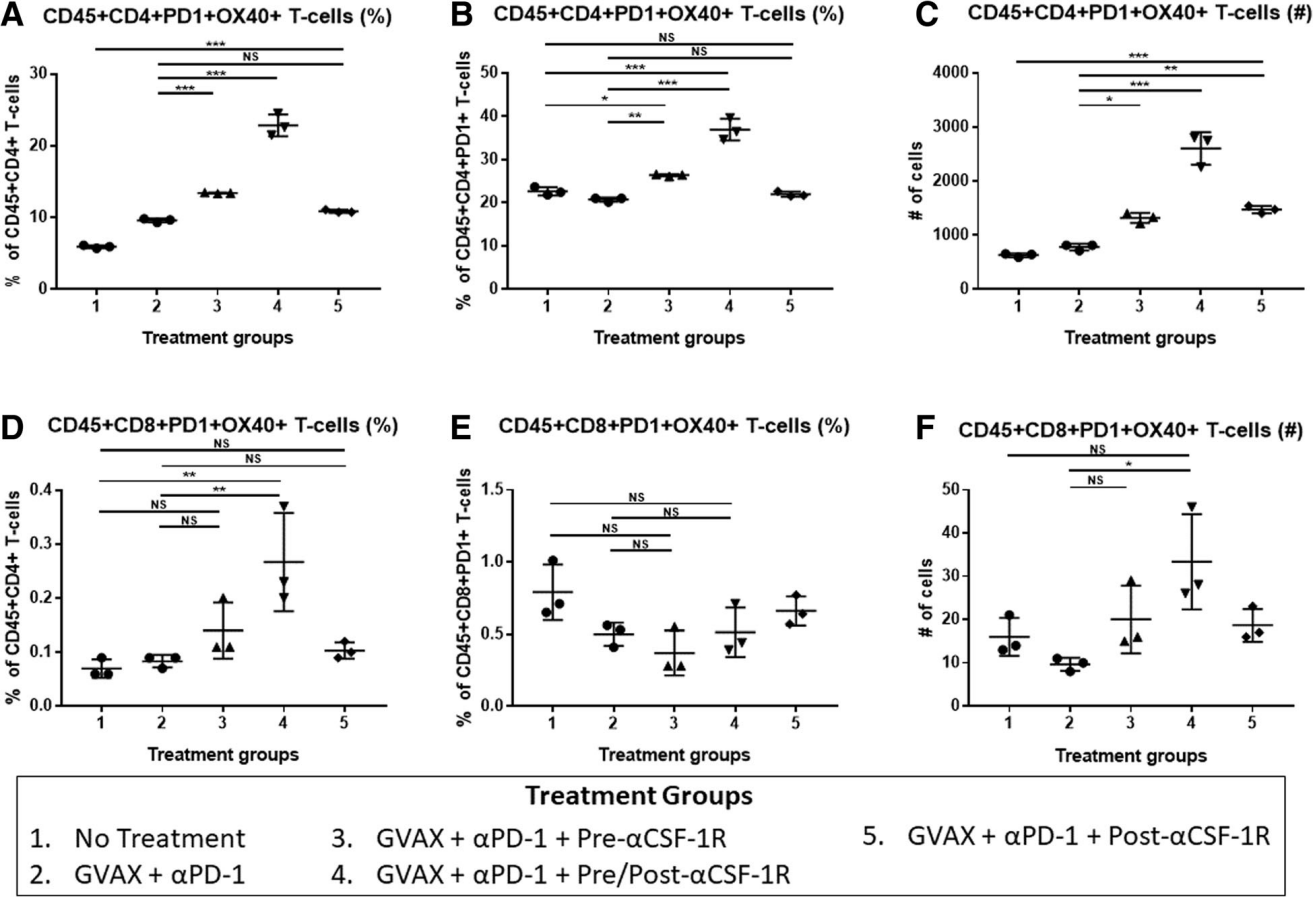
The PD-1 + OX40+ CD4+ T-cells increase with the addition of αCSF-1R to GVAX therapy and αPD-1. Liver metastatic KPC tumor-bearing mice were sacrificed on day 14 after receiving treatment, and flow cytometry analysis was performed on the isolated tumor infiltrating immune cells. The percentage of PD-1 + OX40+ cells within the (**a**) CD45 + CD4+ and (**d**) CD45 + CD8+ T-cell populations. The proportion of cells expressing OX40+ amongst (**b**) CD45 + CD4 + PD-1+ and (**e**) CD45 + CD8 + PD-1+ T-cells. The number of (**c**) CD45 + CD4 + PD-1 + OX40+ and (**f**) CD45 + CD8+ PD-1 + OX40+ T-cells. *N* = 3 for each treatment group, and the isolated immune cells from mice from the same treatment group were pooled and measured in triplicates. *p < 0.05; **p < 0.01; ***p < 0.001; NS, non-significant

To investigate if the addition of αCSF-1R affects other markers of T-cell activation, we also studied OX40 expression in the flow cytometry analysis described above. The addition of αCSF-1R both before and after GVAX vaccination in the “GVAX + αPD-1 + Pre/Post-αCSF-1R” group also increased the number and percentage of PD-1 + OX40+ co-expressing CD4+ T-cells compared to the control groups, non-treatment and GVAX + αPD-1 treatment (Fig. 4a-c). Up to 40% of PD-1+ CD4+ T-cells expressed OX40+ in the “GVAX + αPD-1 + Pre/Post-αCSF-1R” group, which is about 2 times higher than the non-αCSF-1R containing groups (Fig. 4b). OX40+ expression on CD8+ T-cells was low in all experimental groups, and as such, co-expression of PD-1 and OX40 was low (Fig. 4d-f). Nonetheless, the percentage of PD-1 + OX40+ T-cells amongst CD8+ T-cells was a higher in the “GVAX + αPD-1 + Pre/Post-αCSF-1R” group compared to control treatments, non-treatment and GVAX + αPD-1 groups, whereas there was no statistically significant difference between the “GVAX + αPD-1 + Pre-αCSF-1R” and “GVAX + αPD-1 + Post-αCSF-1R” and the control groups. Amongst PD-1+ CD8+ T-cells however, this slight increase of PD-1 + OX40+ CD8+ T-cells in the “GVAX + αPD-1 + Pre/Post-αCSF-1R” group was not statistically significant from the control groups.

### Tumor infiltrating CD8 + PD-1 + CD137+ T-cells express IFN-γ

**Fig. 5:**
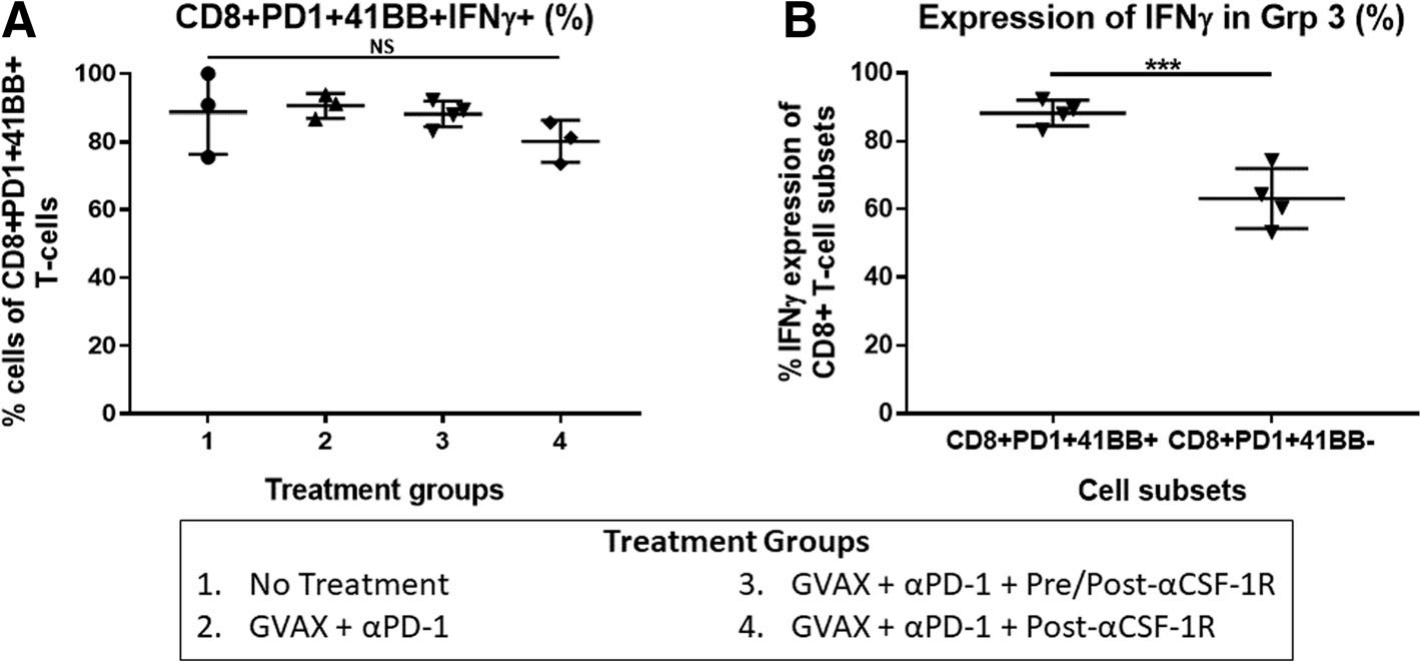
CD8+ T-cells that co-express PD-1 and CD137 express IFN-γ. Liver metastatic KPC tumor-bearing mice were sacrificed on day 14 after receiving treatment, and flow cytometry analysis was performed on the isolated tumor infiltrating CD8+ T-cells after incubation with CD3/CD28 stimulation beads. **a** IFN-γ production of CD8 + PD-1 + CD137+ T-cells. **b** IFN-γ production of CD8 + PD-1 + CD137+ and CD8 + PD-1 + CD137- T-cells in the GVAX + αPD-1 + Pre/Post-αCSF-1R group. *N* = 3–4 for each treatment group, and each data point represents cells from one mouse. The error bars represent mean with standard deviation. *** *p* < 0.001; NS, non-significant

These result suggest that αCSF-1R may convert the PD-1+ exhausted T-cells into activated effector T-cells that express CD137. To examine the activation level of these CD8+ T-cells that co-expressed PD-1 and CD137, we measured the IFN-γ production of these tumor infiltrating CD8 + PD-1 + CD137+ T-cells after isolating CD8+ T-cells and incubating them with CD3/CD28 stimulation beads, and then performed flow cytometry analysis for CD137, PD-1 and IFN-γ expression amongst these CD8+ T-cells. Most of the CD8 + PD-1 + CD137+ T-cells (median about 80–90%) in the treated, as well as untreated groups, expressed IFN-γ (Fig. 5a). Interestingly, a higher percentage of the CD8 + PD-1 + CD137+ T-cells expressed IFN-γ compared to the CD8 + PD-1 + CD137- T-cells (Fig. 5b), suggesting that CD8 + PD-1 + CD137- T-cells, which may express other activation markers, are less effective.

## Discussion

In this report, we found that higher expression of CSF-1R is associated with a higher intratumoral myeloid to lymphoid ratio, which is associated with poorer survival in PDAC patients who received GVAX. Interestingly, this higher CSF-1R expression is associated with a greater intratumoral infiltration of immature DCs, but not mature DCs. As DCs fail to mature and activate T-cells, these T-cells can become tolerogenic or anergic. Hence, low CSF-1R expression leads to higher infiltration of lymphoid cells (including CD8+ T-cells), and lower infiltration of certain myeloid cell subsets associated with poor prognosis, while sparing the mature DCs that are important for antigen presentation in vaccine therapies. These findings suggest that CSF-1R, in conjunction with cancer vaccine therapy, could be targeted to deplete immunosuppressing tumor infiltrating myeloid cells and modulate the remaining myeloid cells towards an anti-tumor phenotype. This study subsequently demonstrated in a preclinical model that anti-CSF-1R antibody therapy enhanced the antitumor activity of the combination of GVAX and anti-PD-1 antibody. The anti-tumor efficacy of anti-CSF-1R antibody appears to be partially achieved by its role in converting PD-1+ exhausted T-cells into CD137+ activated T-cells in the PDAC tumors. Accordingly, we identified a subpopulation within both CD4+ and CD8+ T-cells that expressed both PD-1 and CD137. The increase in expression of CD137 after therapy that included anti-CSF-1R, compared to treatment that did not, was more robust within PD-1+ CD8+ T-cells than PD-1+ CD4+ T-cells. The vast majority of these PD-1 + CD137+ CD8+ T-cells expressed IFN-γ, and thus were functional effector T-cells. Furthermore, combination immunotherapy including anti-CSF-1R also increased OX40 expression amongst PD-1+ CD4+ T-cells, though there was minimal expression of OX40 on PD-1+ CD8+ T-cells.

CD137 and OX40 are members of the tumor necrosis factor receptor (TNFR) superfamily, and are involved in T-cell expansion and prevention of antigen-induced cell death [32]. They are thereby responsible for enlarging the memory T-cell population, which is essential to maintaining the immune protection conferred by vaccinations [33]. It was previously thought that there was a dichotomy in roles between these two members of the TNFR superfamily, such that OX40 played a more central function for CD4+ T-cell expansion and survival, and 41BB played a similar function for CD8+ T-cells, and to some extent, our findings may reflect this. [33–35] However, subsequent studies suggest that these receptors have roles in both cell types, and that their expression is dependent on the immune environment and timing from initial T-cell priming. [36, 37] It has been shown that CD137 expression on CD8+ T-cells persists for a longer time, whereas OX40 expression is transient [37]. As such, it is possible that this combination treatment with anti-CSF-1R either does not induce OX40 expression on PD-1+ CD8+ T-cells, or the expression of OX40 was too transient and subsequently not measured.

The PD-1 + CD137+ T-cells were found in the PDAC tumors of all the mice, regardless of whether they were treated by GVAX, anti-PD-1 or anti-CSF-1R antibody. Although PD1 + CD137+ T-cells are only a small percentage among all the T-cells, it is possible that PD-1+ T-cells expressing other activation markers exist. More importantly, as the majority of the PD-1 + CD137+ T-cells have the capacity of expressing IFN-γ, even if this subpopulation of T-cells is small, they may still possess a strong anti-tumor effector T-cell function. Indeed, a small percentage of CD3 + CD8+ T-cells that are PD-1 + CD137+ (0.6 to 5.1%) have been found in some patients with metastatic melanoma and appear to have the ability to recognize neoantigens [38].

It remains to be explored how anti-CSF-1R antibody leads to the significant increase in the percentage and number of intratumoral PD-1+ CD4+ and PD-1+ CD8+ T-cells that express CD137 and PD-1+ CD4+ T-cells that express OX40. As CD137 expression represents T-cell activation during the T-cell priming process, it is possible that the effect of anti-CSF-1R on myeloid cells accounted for this modulation of T-cell priming. Anti-CSF-1R antibody has been reported to spare the activated antigen-presenting DCs from depletion from the TME [20, 21, 39]. This study supports the rational that targeting tumor infiltrating myeloid cells can activate T-cells with exhausted phenotypes, but the survival advantage of the combination therapy with αCSF-1R is still limited. The limited efficacy is likely partly due CSF-1R being a pan-myeloid marker, and thus a more specific agent targeting immunosuppressive myeloid cells may improve the efficacy.

Interestingly, the sequence of anti-CSF-1R antibody and the treatment of anti-PD-1 antibody and GVAX appears to be critical. Having anti-CSF1R antibody both before and after the GVAX treatment yields the largest survival benefit and the largest increase in PD-1 + CD137+ T-cells and PD-1 + OX40+ CD4+ T-cells. However, in human studies, such a combination of anti-PD-1 antibody, GVAX, and anti-CSF-1R antibody would be administered as multiple cycles. When they are given for multiple cycles, the differential effect of the sequencing of anti-CSF-1R with anti-PD-1 and GVAX treatment may be abrogated, and the effects of multiple cycles of therapy will be investigated in future studies. Nevertheless, anti-tumor efficacy of the combination of anti-PD-1 antibody, GVAX and anti-CSF1R antibody warrants further investigation in human studies for PDAC.

## Conclusions

In this study, we demonstrate that the level of expression of CSF-1R within the lymphoid aggregates induced by GVAX affects the tumor immune microenvironment; high CSF-1R expression is associated with an immunosuppressive environment in patients who received GVAX. To counteract the immunosuppressing effects of CSF-1R expression within the TME, we examined the combination therapy of GVAX vaccine, and anti-PD-1 and anti-CSF-1R antibodies in a murine model of metastatic PDAC. This combination therapy improved the survival rate, increased the percentage of CD4+ and CD8+ T-cells that co-expressed PD-1 and CD137 and increased the percentage of PD-1 + OX40+ CD4+ T-cells within the tumors. The PD-1 + CD137+ CD8+ T-cells had high IFN-γ expression, suggesting that these cells in part were responsible for the improved anti-tumor activity of the combination therapy. Our results demonstrate that adding a myeloid-targeting agent to vaccine-based cancer immunotherapy can reverse the anergy of intra-tumoral T-cells in immune-quiescent tumors. The findings in this study support the clinical evaluation of this combination therapy in patients with PDAC.

## Additional files

Additional file 1: **Figure S1.** PD-1 expression on CD4+ and CD8+ T-cells can increase with the addition of αCSF-1R to GVAX therapy and αPD-1. Liver metastatic KPC tumor-bearing mice were sacrificed on day 14 after receiving treatment, and flow cytometry analysis was performed on the isolated tumor infiltrating immune cells. PD-1 expression within the (A) CD45 + CD4+ T-cell and (C) CD45 + CD8+ T-cell populations. The number of (B) CD45 + CD4 + PD-1+ and (D) CD45 + CD8 + PD-1+ T-cells. *N* = 3 for each treatment group, and the cells from the mice from the same treatment group were pooled and measured in triplicates. * *p* < 0.05; ** *p* < 0.01; *** *p* < 0.001; NS, non-significant. (PNG 32 kb)

Additional file 2: **Figure S2.** PD-1 + CD137+ expression increases when αCSF-1R is administered before and after GVAX vaccination in combination with αPD-1. Representative flow cytometry dot plots of PD-1 and CD137 expression amongst CD8+ and CD4+ T-cells between the different treatment regimens containing αCSF-1R, GVAX and αPD-1. (PNG 269 kb)

## Abbreviations

CI: Confidence interval
CSF-1: Colony stimulating factor-1
CSF-1R: Colony stimulating factor-1 receptor
DC: Dendritic cell
DC-SIGN: Dendritic Cell-Specific Intercellular adhesion molecule-3-Grabbing Non-integrin
GM-CSF: Granulocyte-macrophage colony-stimulating factor
HR: hazard ratio
IHC: Immunohistochemistry
MDSC: Myeloid-derived suppressor cell
PD-1: Programmed death-1
PDAC: Pancreatic ductal adenocarcinoma
TAM: Tumor-associated macrophage
TME: Tumor microenvironment
TNFR: Tumor necrosis factor receptor
αCSF-1R: Anti-colony stimulating factor-1 receptor antibody
αPD-1: Anti-programmed death-1 antibody
