# Supplementary figures and images for: Targeting myeloid-inflamed tumor with anti-CSF-1R antibody expands CD137+ effector T-cells in the murine model of pancreatic cancer
Source: J Immunother Cancer. 2018 Nov 13;6:118. doi: 10.1186/s40425-018-0435-6 (PMC6234697; doi:10.1186/s40425-018-0435-6)

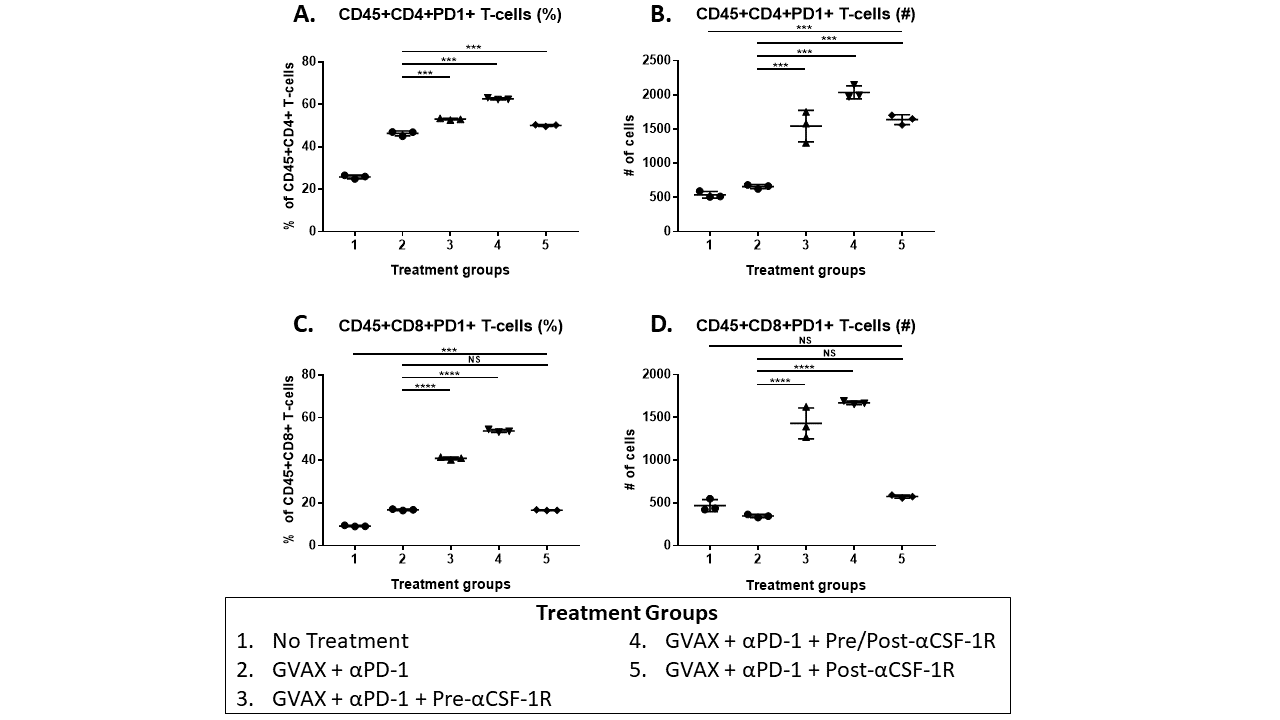

Supplement: Supplementary file 1 — Figure S1. PD-1 expression on CD4+ and CD8+ T-cells can increase with the addition of αCSF-1R to GVAX therapy and αPD-1. Liver metastatic KPC tumor-bearing mice were sacrificed on day 14 after receiving treatment, and flow cytometry analysis was performed on the isolated tumor infiltrating immune cells. PD-1 expression within the (A) CD45 + CD4+ T-cell and (C) CD45 + CD8+ T-cell populations. The number of (B) CD45 + CD4 + PD-1+ and (D) CD45 + CD8 + PD-1+ T-cells. N = 3 for each treatment group, and the cells from the mice from the same treatment group were pooled and measured in triplicates. * p < 0.05; ** p < 0.01; *** p < 0.001; NS, non-significant. (PNG 32 kb) [file 40425_2018_435_MOESM1_ESM.png]

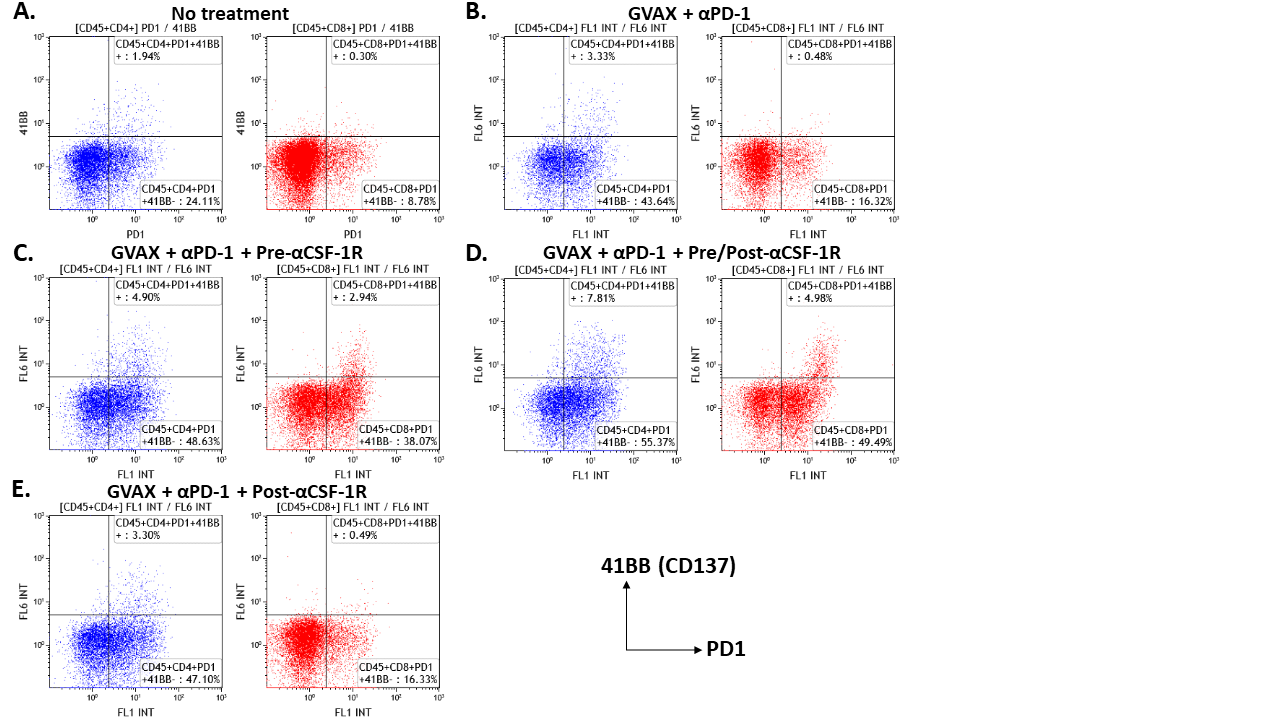

Supplement: Supplementary file 2 — Figure S2. PD-1 + CD137+ expression increases when αCSF-1R is administered before and after GVAX vaccination in combination with αPD-1. Representative flow cytometry dot plots of PD-1 and CD137 expression amongst CD8+ and CD4+ T-cells between the different treatment regimens containing αCSF-1R, GVAX and αPD-1. (PNG 269 kb) [file 40425_2018_435_MOESM2_ESM.png]
